# Overall survival in patients with re-excision of positive microscopic margins of limb and trunk wall soft tissue sarcoma operated outside of a reference center: a nationwide cohort analysis

**DOI:** 10.1186/s12885-022-10121-5

**Published:** 2022-10-03

**Authors:** Francois Gouin, Eberhard Stoeckle, Charles Honoré, Mickael Ropars, Mehrdad Jafari, Jean Camille Mattei, Alexandre Rochwerger, Sébastien Carrere, Denis Waast, Gwenaël Ferron, Jean-Christophe Machiavello, Philippe Anract, Frédéric Marchal, François Sirveaux, Oren Marco, Jérôme Guiramand, Brice Paquette, Antonio Di Marco, Sylvain Causeret, Jean-Marc Guilloit, Pauline Soibinet, Dimitri Tzanis, Pierre Gimbergues, Fabrice Fiorenza, Franck Dujardin, Louis R. Le Nail, Jean-Christophe Ruzic, Claire Chemin-Airiau, Magali Morelle, Pierre Meeus, Marie Karanian, François Le Loarer, Gualter Vaz, Jean-Yves Blay, on behalf of the NETSARC/RREPS and French Sarcoma Group-Groupe d’Etude des Tumeurs Osseuses (GSF-GETO) networks

**Affiliations:** 1 Surgery department, Centre Léon Bérard, Lyon, France; 2 Surgery department, Institut Bergonié, Bordeaux, France; 3 Surgery department, Gustave Roussy Cancer Campus, Villejuif, France; 4 Orthopedic surgery department, CHU de Rennes, Rennes, France; 5 General and digestive oncologic surgery, Centre Oscar Lambret, Lille, France; 6 Ramsay Santé, Hôpital Privé Clairval, Marseille, France; 7 Aix Marseille University, Marseille, France; 8 INSERM, MMG, Marseille, France; 9 Orthopedic and traumatologic surgery department, Hôpital Nord, Marseille, France; 10 Hopital de la Conception, APHM, Marseille, France; 11 Surgery department, Institut de recherche en cancérologie, Montpellier, France; 12 Orthopedic and traumatologic surgery clinic, CHU, Nantes, France; 13 Surgery department, Toulouse Oncopole, Toulouse, France; 14 Senology surgery department, Onco-gynécologique et Reconstructrice, Centre Antoine Lacassagne, Nice, France; 15 Orthopedic surgery department, Hôpital Cochin, AP-HP, Paris, France; 16 Surgery department, Institut de Cancérologie de Lorraine, Université de Lorraine, CNRS, CRAN, UMR 7039, Vandoeuvre-les-Nancy, France; 17 Orthopedy department, CHU de Nancy, Nancy, France; 18 Reconstructive et esthetic plastic surgery, Hôpital Saint Louis, Paris, France; 19 Surgery department, Institut Paoli Calmette, Marseille, France; 20 Department of Digestive Surgery, Jean Minjoz University Hospital, Besançon, France; 21 Orthopedic surgery department, CHU de Strasbourg, Strasbourg, France; 22 Surgery department, Centre George-François Leclerc, Dijon, France; 23 Visceral et digestive surgery department, Centre François Baclesse, Caen, France; 24 Medical Oncology department, Institut J Godinot, Reims, France; 25 Surgery department, Institut Curie, PSL university, Paris, France; 26 Surgery department, Centre Jean Perrin, Clermont Ferrand, France; 27 Orthopedic and traumatology surgery department, CHU Limoges, Limoges, France; 28 Medical Oncology and Surgical Oncology department, Centre Henri Becquerel, Rouen, France; 29 Onco-orthopedic surgery department, Hôpital Trousseau, CHRU de Tours, Tours, France; 30 Orthopedic surgery, CHU St-Pierre, St Pierre, La Réunion France; 31 Clinical research and innovation department, Centre Léon Bérard, Lyon, France; 32 Department of Biopathology, Centre Léon Bérard, Lyon, France; 33 Anatomo-pathology surgery department, Institut Bergonié, Bordeaux, France; 34 Medical oncology Centre Léon Bérard, Lyon, France; 35 University Claude Bernard Lyon I, Lyon, France; 36 Headquarters, Unicancer, Paris, France

**Keywords:** Soft tissue sarcoma, Surgery, Relapse, Reference center, Multidisciplinary tumor board resection margins, Survival

## Abstract

**Background:**

This French nationwide NETSARC exhaustive prospective cohort aims to explore the impact of systematic re-excision (RE) as adjuvant care on overall survival (OS), local recurrence free survival (LRFS), and local and distant control (RFS) in patients with soft tissue sarcoma (STS) with positive microscopic margins (R1) after initial resection performed outside of a reference center.

**Methods:**

Eligible patients had experienced STS surgery outside a reference center from 2010 to 2017, and had R1 margins after initial surgery. Characteristics and treatment comparisons used chi-square for categorical variables and Kruskall-Wallis test for continuous data. Survival distributions were compared in patients reexcised (RE) or not (No-RE) using a log-rank test. A Cox proportional hazard model was used for subgroup analysis.

**Results:**

A total of 1,284 patients had experienced initial STS surgery outside NETSARC with R1 margins, including 1,029 patients with second operation documented. Among the latter, 698 patients experienced re-excision, and 331 were not re-excised. Characteristics were significantly different regarding patient age, tumor site, tumor size, tumor depth, and histotype in the population of patients re-excised (RE) or not (No-RE). The study identified RE as an independent favorable factor for OS (HR 0.36, 95%CI 0.23–0.56, *p*<0.0001), for LRFS (HR 0.45, 95%CI 0.36–0.56, *p*<0.0001), and for RFS (HR 0.35, 95%CI 0.26–0.46, *p*<0.0001).

**Conclusion:**

This large nationwide series shows that RE improved overall survival in patients with STS of extremities and trunk wall, with prior R1 resection performed outside of a reference center. RE as part of adjuvant care should be systematically considered.

Level of evidence II

**Supplementary Information:**

The online version contains supplementary material available at 10.1186/s12885-022-10121-5.

## Introduction

Soft tissue sarcomas (STS) are a heterogenous group of malignant tumors gathering over 155 histotypes and molecular subtypes that constitute approximatively 1% of all malignancies [1]. Extremities and trunk-wall locations are the most frequent location of STS [2]. En-bloc surgical resection with clear margins (R0) after review by a multidisciplinary tumor board (MDTB) in an expert center is the mainstay of curative treatment [3]. Nevertheless even in expert centers, this objective is not always achievable for all patients: positive microscopic margins (R1) are reported in 16 to 34% of the cases in specialized centers [4–6] and in up to 70% in non-specialized centers [7].

In case of unplanned macroscopically complete resection outside of a specialized center, re-excision (RE) followed by radiotherapy is generally considered [3, 8]. Most studies reported that RE improves local tumor control, and better local and distant relapse free survival (LRFS) [5, 9–13]. Whether RE impact overall survival (OS) is conversely debated, raising the issue of surveillance measures or a more aggressive approach with systematic RE. Based on the indirect correlation between re-excision of residual tumor in tumor bed and progression and/or survival, several studies reported that patients benefit from RE [5, 9–12] after unplanned resection while others did not evidence that residual tumor in tumor bed re-excision was associated with improved disease specific survival,[13] distant metastasis risk, and overall survival [14] or reported similar overall survival (OS) in patients with unplanned initial resection re-operated or not [15].

While patients operated in high-volume multidisciplinary sarcoma centers have better outcome [16], many patients are initially operated out of a sarcoma center and the question of systematic re-operation is still debated, considering the inconsistent impact of RE on overall survival across series. We used the French nationwide prospective database NETSARC to assess overall survival (OS), local and distant relapse free survival (RFS), and local control (LRFS) in patients with R1 margins who had been operated for a trunk wall and limb soft tissue sarcoma outside of a reference center who experienced re-excision (RE) or not (no-RE).

## Material and patients

### Objectives

This study aims to assess the overall survival in patients with STS of the extremities or the trunk wall, who experienced initial surgery with R1-margins, performed outside of the French nationwide NETSARC reference centers, reexcised (RE) or not (No-RE). Secondary objectives included local and distant relapse free survival (RFS) and local recurrence free survival (LRFS). The data-collection and analysis received approval from the national Advisory Committee on Information Processing in Health Research (*Comité consultatif sur le traitement de l’information en matière de recherche dans le domaine de la santé*, *CCTIRS)* n°10.403, September 16, 2010, and from the French data protection authority (*Commission Nationale Informatique et Liberté, CNIL),* n° 910390, July 15, 2013.

### Patients’ selection

**Fig. 1:**
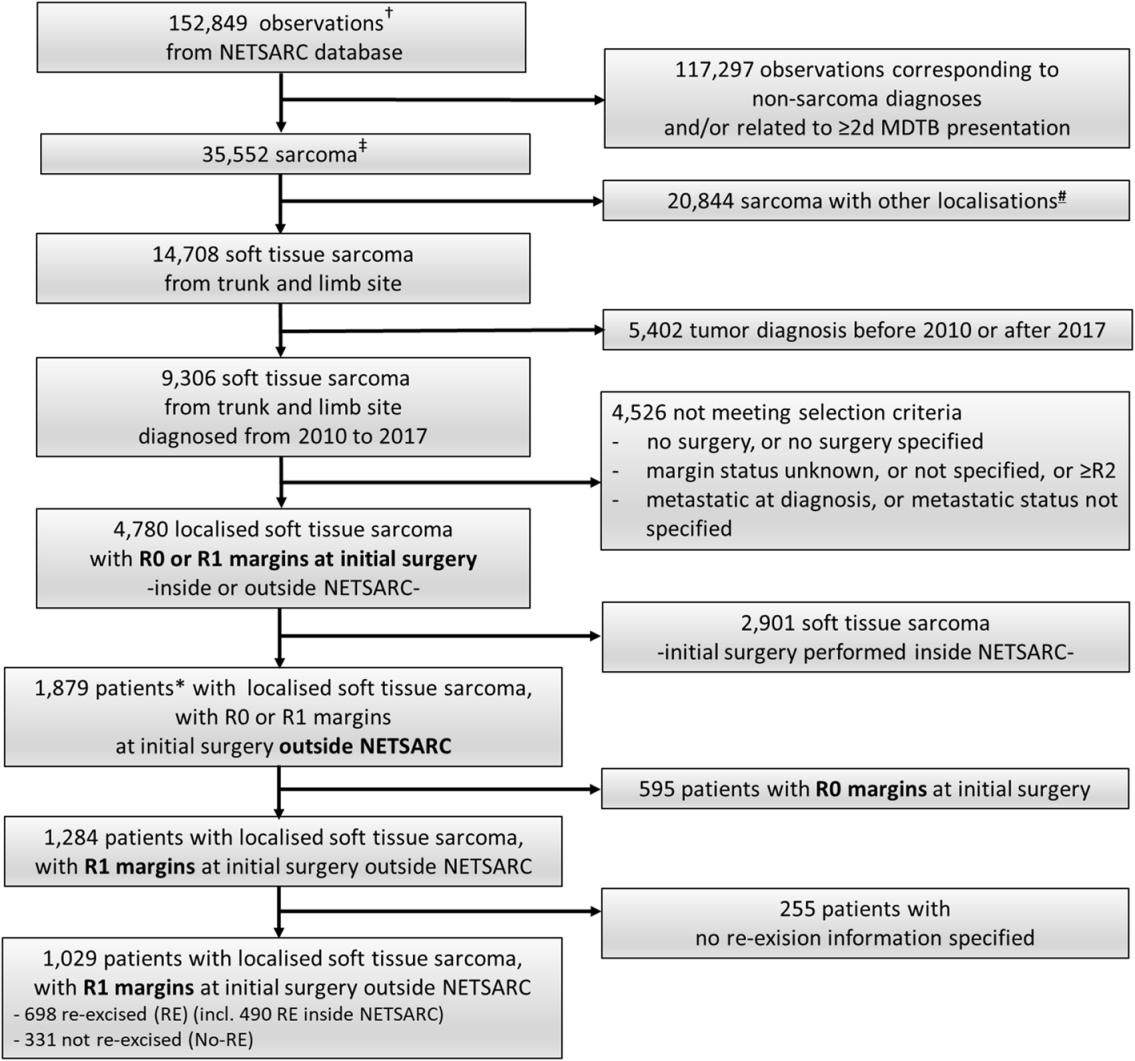
Flow-chart. The statistical unit is successively ^†^observations reviewed by the MDTB, ^‡^tumor, ^*^patient. ^#^Other localizations include bone (*n*=5,882); viscera (*n*=6,034); head and neck (*n*=2,235); internal trunk (*n*=6.562); soft tissue (*n*=116); unknown (*n*=15); MDTB: multi-disciplinary tumor board

The study enrolled patients with localized STS of trunk and limbs prospectively registered in the NETSARC database between 07/2010 and 12/2017, with specified R1 margins after initial surgery performed outside a NETSARC reference center. Desmoid, well differentiated (atypical lipomatous tumors), dermato-fibrosarcoma protuberans were excluded because they are rarely life-threatening diseases. Patients with metastasis at diagnosis or unknown initial metastatic status were also excluded (Fig. 1).

The affiliation of the first surgeon was collected and categorized within or outside a NETSARC reference center; patients were considered as operated in a NETSARC reference center if the surgeon was registered in NETSARC network (https://NetSarc.sarcomabcb.org), and conversely, as operated in a non-expert center if the surgeon was not referenced in the NETSARC network.

### NETSARC network and database

The French nationwide reference network for clinical and pathological sarcoma care NETSARC supported by the French Institute of Cancer (INCa) set up a nationwide database currently considered to be close to exhaustivity of all STS in France [2]. All sarcoma including suspicion for sarcoma are presented and reviewed by a multidisciplinary tumor board (MDTB) involving the 26 French cancer centers and registered at first presentation in a database by a dedicated team of clinical research assistants, at any time of the disease course (before diagnosis, before any treatment, after primary surgery, before adjuvant therapy, at the date of oncologic event or/and clinical trial screening).

In France, for each operated patient regardless of the institution, a centralized review with double-interpretation is deemed mandatory and pathological reports encourage the clinicians to present each case to MDTB. Thus, data from patients operated in or outside of NETSARC reference center network are collected in NETSARC database.

The database includes patient and tumor characteristics, surgery, relapse and survival. The wider tumor diameter defined tumor size. The *National Federation of Cancer Centres* (FNCLCC, Unicancer) specified 4 categories for histological grades: grade 1, 2, 3, and ungraded tumors. Sarcomas without grading resulted from histology grading failure or lack of critical elements to complete the diagnosis, as determined by experts.

The quality of surgical resection used the definition of the *Union Internationale Contre le Cancer* (UICC) [17], and margin status determination is based on pathology and surgery reports when available: R0 referred to clear margins – in the present study R0 margins qualified a *monobloc* resection and clear margins specified on pathological report; R1 margins referred to (possible) microscopic residual disease, with visible tumor cells on resection margins (positive microscopic margins) – in the present study R1 margins indicated margins not confirmed as R0 or R2. R2 resulted from fragmented resections, or operative/pathological reports suggesting or notifying macroscopic residual tumor and/or fragmented resection; cases with no margin characterization were excluded (missing data) (Fig. 1). Patients referred after first surgery, with any residual tumor, hematoma, and scar track are generally examined by magnetic resonance imaging and identification of all pathologic features (diagnosis and margins), primary surgical procedure, pre- (if available) and post-operative imaging, and patient general assessment are performed.

### Statistical method

Qualitative variables were described with frequencies and percentages, and quantitative variables with average and range. Comparisons between groups used the chi-square test for qualitative variables and Kruskall-Wallis test for quantitative variables.

The diagnosis date was the date of pathological diagnosis (biopsy or first surgery). Overall survival (OS) was defined as the time from the date of diagnosis to the date of the last follow-up or death due to any cause. Local and/or distant relapse free survival (RFS) was defined as the time from the date of diagnosis to the date of last follow-up or the date of first local progression, metastatic progression, or death, whichever occurred first. Local relapse free survival (LRFS) was computed from the diagnosis date to the date of last follow-up or the date of first local progression. OS and RFS were calculated using the Kaplan-Meier method. Duration of follow-up was estimated using the reverse Kaplan-Meier method and expressed with Q1-Q3 interval. Survival distributions were compared between groups using the Log–rank test and the multivariate analysis used the Cox proportional hazard model. Competing events to local recurrence are considered in a competing risk approach to estimate LRFS. The cumulative incidence function and non-parametric Gray’s test were used to estimate and to compare cumulative incidence function between the groups. Univariate and multivariate analysis explored whether first resection outside NETSARC impacted OS, RFS, and LRFS in R1 patients. Multivariate analysis used a Fine-Gray model [18], and included usual prognostic factors for sarcoma.

Sub-group analyses explored whether RE may benefit to specific subgroups of patients.

RE status was not available for 255 patients with R1 margins. Considering that all re-excision of R1 patients were carried out in NETSARC reference centers, missing data regarding RE status were considered as missing not at random. Characteristics of patients with no specified margin status are presented in Supplementary material S1 and a sensitivity analysis was performed considering these patients as not reoperated in Supplementary material S2.

A propensity score matching analysis was carried out and presented in supplementary material S3.

The cut-off date for data analysis was 2020, Novembre 9. Analyses were performed using SAS version 9.4 software (SAS Institute Inc., Cary, NC, USA) and significance for all statistical tests was evaluated using two-sided *p* values.

## Results

### Patients’ characteristics

Among the 1284 (68.3%) patients operated outside NETSARC centers with specified R1 margins at initial surgery, a total of 1029 had re-excision information available, 698 patients were re-excised (RE) and 331 patients had no re-excision (No-RE) (Fig. 1, Table 1)

**Table 1:** Characteristics of R1 patients first operated outside NETSARC reference centers (*n*=1,029). Data are mean (SD) or n (%). MDTB: MultiDisciplinary Tumor Board. Percentages might not add up to 100% due to rounding. KW: Kruskal-Wallis

	R1 patients non re–excised (noRE), *n* =331	R1 patients re–excised (RE), *n* =698	R1 patients re–excised (RE) or not (NoRE) *n*= 1,029	*p* value for R1 patients noRE *versus* RE
Gender				
Female	147 (44%)	313 (45%)	460 (44.7%)	Chi-2 *p* =0.897
Male	184 (56%)	385 (55%)	569 (55.3%)	
Age at diagnostic	59.5 (21.2)	57.1 (18.3)	57.88 (19.31)	Chi 2 *p* =0.011
Site of the tumor				KW *p* =0.020
Trunk wall	134 (41%)	206 (30%)	445 (43.2%)	
Upper limb	62 (19%)	182 (26%)	340 (33.0%)	
Lower limb	135 (41%)	310 (44%)	244 (23.7%)	
Size of the tumor (mm)	72.8 (58.5)	47.9 (40.5)	55.75 (48.3)	KW *p* <0.001
Depth of the tumor			45 (5-500)	Chi-2 *p* <0.001
Superficial (Sus–facia)	89 (29%)	303 (46%)	392 (40.9%)	
Deep (Sub-facia)	214 (71%)	352 (54%)	566 (59.1%)	
Histology				
Liposarcoma	62 (19%)	85 (12%)	147 (14.3%)	Chi-2 *p* =0.001
Synovial sarcoma	21 (6%)	32 (5%)	53 (5.2%)	
Leiomyosarcoma	48 (14%)	160 (23%)	208 (20.2%)	
Miscellaneous sarcomas	46 (14%)	76 (11%)	122 (11.9%)	
Myxofibrosarcoma	38 (11%)	115 (16%)	153 (14.9%)	
Undifferentiated pleomorphic sarcoma	78 (24%)	153 (22%)	231 (22.4%)	
Other	38 (11%)	77 (11%)	115 (11.2%)	
Grade of the tumor				
Grade 1	43 (14%)	101 (15%)	144 (15.1%)	Chi-2 *p* =0.106
Grade 2	81 (27%)	227 (35%)	308 (32.4%)	
Grade 3	98 (33%)	193 (29%)	291 (30.6%)	
Non dimmable	74 (25%)	135 (21%)	209 (22.0%)	
MDTB before treatment				
No	286 (86%)	642 (92%)	928 (90.2%)	Chi-2 *p* =0.005
Yes	45 (14%)	56 (8%)	101 (9.8%)	
2^d^ surgery performed				
Inside NETSARC	–	490 (70%)	490 (47.6%)	
Outside NETSARC	–	175 (25%)	175 (17.0%)	
Unkwown	–	33 (5%)	33 (3.2%)	
Quality of resection				
R0	0 (0%)	599 (86%)	599 (60.4%)	Chi-2 *p* <0.001
R1	331(100%)	42 (6%)	373 (37.6%)	
Margins not evaluable	0 (0%)	2 (0%)	2 (0%)	
Not specified	0 (0%)	55 (8%)	55 (5.3%)	

The characteristics of RE and no-RE patients were significantly different regarding mean age at diagnosis (*p* =0.011), tumor sites (trunk wall, upper limb, lower limb, *p* =0.002), tumor size (*p* <0.001), tumor depth (*p* <0.001) and histotype (*p*=0.001)

### Impact of RE on overall survival (OS) (Fig. 2A, Table 2)

The median follow-up between the two groups was similar (RE: 32.95, 9.1-59.5; No-RE: 30.58, 7.52-63.24 months). In univariate analysis, RE was associated with an improved OS (HR 0.33, 95%CI 0.22–0.49), *p*<0.0001). OS also significantly correlated with age at diagnosis (*p*<0.0001), tumor size (p <0.0001), tumor grade (*p*=0.00), histotype (*p*=0.03), and tumor location (*p*=0.02). The multivariate analysis identified RE as an independent favorable prognostic factor for OS (HR 0.36, 95%CI 0.23–0.56, *p*<0.0001), along with age at diagnosis, and tumor size, site, grade, and histotype (Table 2).

**Fig. 2:**
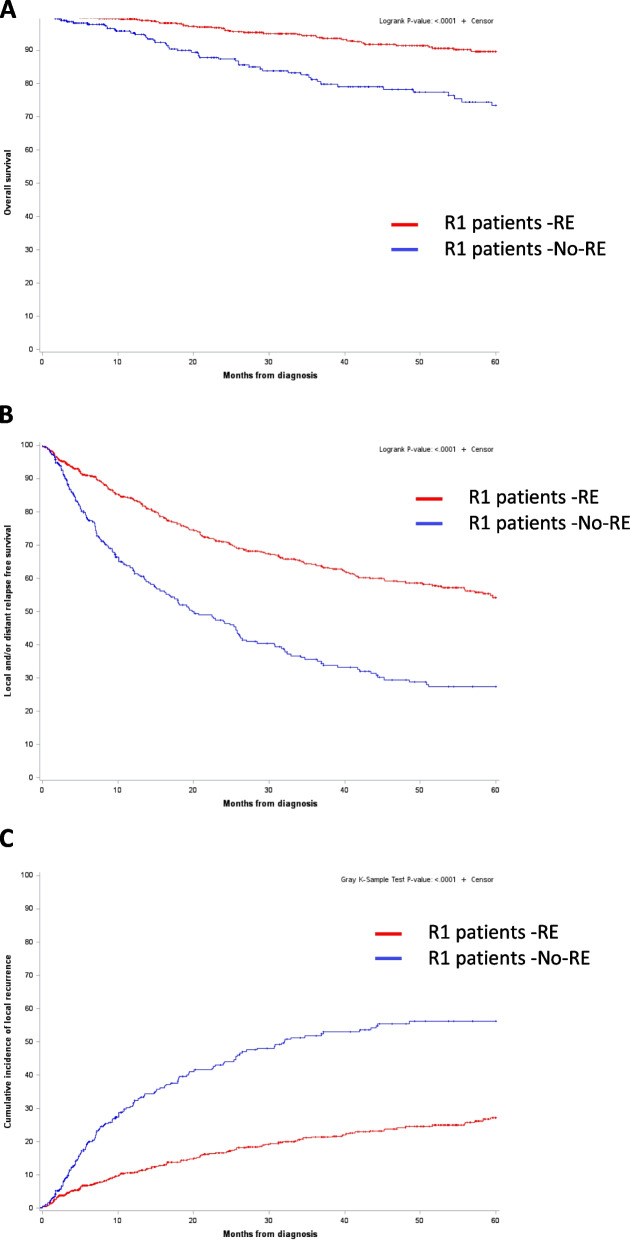
Overall survival (OS)(A), local and/or distant relapse free survival (RFS) (B) and cumulative incidence of local recurrence (C) in patients with R1 resection outside NETSARC reference centers, for whom secondary resection (RE) was performed or not (No-RE) (*n*=1,029).

**Table 2:** Univariate and multivariate analysis for overall survival, relapse free survival, and local recurrence free survival of patients first operated outside NETSARC center with R1 margins (*n*=1,029 patients)

	Overall Survival		Local and/or distant Relapse Free Survival		Local Recurrence Free Survival	
	Unadjusted HR	Adjusted HR	Unadjusted HR	Adjusted HR	Unadjusted HR	Adjusted HR
Age at diagnosis	1.03 (1.02–1.04**); <.0001**	1.03 (1.01–1.04)**; 0.00**	1.02 (1.02–1.03); **<.0001**	1.02 (1.01–1.03); <.**0001**	1.02 (1.01–1.03); <**.0001**	1.01 (1.00–1.02**); 0.00**
Gender female (ref: male)	0.84 (0.55–1.26); 0.39	0.79 (0.51–1.23); 0.29	0.89 (0.73–1.09); 0.25	0.90 (0.73–1.12); 0.35	1.01 (0.80–1.29); 0.90	1.07 (0.82–1.40); 0.61
Size of the tumor (mm)	1.01 (1.00–1.01); **<.0001**	1.00 (1.00–1.01); **0.01**	1.01 (1.00–1.01)**; <.0001**	1.00 (1.00–1.01); <.**0001**	1.00 (1.00–1.01); <.**0001**	1.00 (1.00–1.01); **0.03**
Site of tumor						
Trunk wall (ref: lower limb)	1.14 (0.74–1.76); 0.55	0.99 (0.62–1.58); 0.95	1.17 (0.93–1.46); 0.18	1.08 (0.84–1.38); 0.55	1.09 (0.82–1.43); 0.56	1.02 (0.75–1.39); 0.89
Upper limb (ref: lower limb)	0.44 (0.23–0.86); **0.02**	0.48 (0.24–0.96); **0.04**	0.85 (0.65–1.10); 0.21	0.81 (0.61–1.08); 0.16	0.87 (0.63–1.20); 0.39	0.86 (0.60–1.23); 0.40
Depth of tumor (ref: superficial)	1.14 (0.74–1.76); 0.56	0.84 (0.51–1.37); 0.48	1.21 (0.98–1.50); 0.08	1.05 (0.83–1.33); 0.67	1.15 (0.89–1.48); 0.29	1.07 (0.80–1.43); 0.67
Grade						
Grade 3 (ref: grades 1–2)	2.27 (1.43–3.66**); 0.00**	1.80 (1.09–2.96); **0.02**	2.04 (1.62–2.56); **<.0001**	1.71 (1.33–2.20); <.**0001**	1.44 (1.08–1.91); **0.01**	1.19 (0.87–1.64); 0.26
Non dimmable (ref: grades 1–2)	1.01 (0.56–1.83); 0.96	0.69 (0.24–1.99); 0.49	1.08 (0.82–1.43); 0.57	1.22 (0.71–2.09); 0.47	1.04 (0.75–1.45); 0.81	1.47 (0.81–2.66); 0.21
MDTB before treatment (ref: no)	1.27 (0.69–2.32); 0.45	1.43 (0.74–2.74); 0.28	1.04 (0.75–1.43); 0.83	0.93 (0.66–1.32); 0.69	0.85 (0.55–1.30); 0.45	0.72 (0.43–1.19); 0.20
Histology						
Leiomyosarcoma (ref: other)	0.38 (0.16–0.91); **0.03**	0.26 (0.09–0.80); **0.02**	0.62 (0.42–0.90); **0.01**	0.66 (0.38–1.16); 0.15	0.60 (0.38–0.96); **0.03**	0.82 (0.42–1.63); 0.58
Liposarcoma (ref: other)	0.58 (0.26–1.29); 0.18	0.22 (0.08–0.63); **0.00**	0.57 (0.38–0.85); **0.01**	0.36 (0.20–0.63); **0.00**	0.67 (0.42–1.07); 0.09	0.60 (0.33–1.10); 0.10
Miscelaneous sarcomas (ref: other)	0.65 (0.29–1.45); 0.29	0.69 (0.25–1.88); 0.47	0.56 (0.37–0.85); **0.01**	0.59 (0.37–0.95); **0.03**	0.47 (0.27–0.80); **0.01**	0.47 (0.26–0.85); **0.01**
Myxofibrosarcoma (ref: other)	0.70 (0.32–1.54); 0.38	0.35 (0.12–0.98); **0.04**	0.99 (0.69–1.43); 0.97	0.83 (0.48–1.44); 0.51	1.08 (0.70–1.67); 0.71	1.25 (0.65–2.41); 0.49
Synovial sarcoma (ref: other)	0.78 (0.30–2.05); 0.61	0.95 (0.29–3.15); 0.93	0.52 (0.31–0.90); **0.02**	0.70 (0.34–1.42); 0.32	0.36 (0.17–0.78); **0.01**	0.46 (0.17–1.23); 0.12
Undiffferentiated sarcoma (ref: other)	1.35 (0.71– 2.58); 0.36	0.66 (0.27–1.63); 0.37	1.09 (0.78–1.52); 0.61	0.77 (0.46–1.31); 0.34	0.91 (0.59–1.39); 0.66	0.91 (0.48–1.71); 0.76
Re–excision (ref: no)	0.33 (0.22– 0.49); **<.0001**	0.36 (0.23–0.56); **<.0001**	0.43 (0.35–0.52); <**.0001**	0.45 (0.36–0.56); <.**0001**	0.35 (0.27–0.44); <.**0001**	0.35 (0.26–0.46) ; <.**0001**

Hazard ratio (HR) (95%CI); *p* value

*MDTB* MultiDisciplinary Tumor Board

### Impact of RE on local and/or distant Relapse Free Survival (RFS) (Fig. 2B)

RFS was significantly better in RE patients in univariate analysis (HR 0.43 95%CI 0.35–0.52, *p*<0.0001) and significantly associated with lower age at diagnosis, smaller size and depth of the tumor, lower grade, and histotype. The multivariate analysis showed that RE was independently associated with a better RFS (HR 0.45 95%CI 0.36–0.56, *p*<0.0001) along with age at diagnosis, tumor size, grade, and histotype (Table 2).

### Impact of RE on Local Recurrence Free Survival (LRFS) (Fig. 2C)

The univariate analysis showed that patients with a first R1 resection outside NETSARC, and re-excision had a significantly better LRFS (HR 0.35, 95%CI 0.27–0.44, *p*<0.0001). Age at diagnosis, tumor size, grade and histotype associated with LRFS (Table 2). Multivariate analysis showed a significantly better LRFS for RE patients (HR 0.35, 95%CI 0.26–0.46, *p*<0.0001). Age at diagnosis, tumor size, and histotype significantly associated with LRFS (Table 2).

### Sub-group analysis survival

**Fig. 3:**
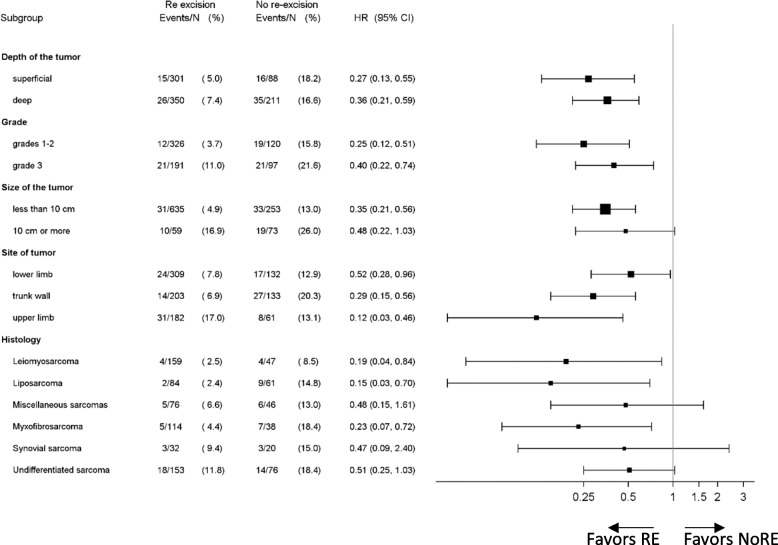
Subgroup analysis and patient overall survival. (unadjusted hazard ratios (HR), with upper CI limit below 1 favors secondary resection (RE) and lower CI limit above 1 favors no secondary resection (NoRE) (*n*=1,029)

A sub-group analysis explored the potential benefit of RE in specific patient subgroups. RE is associated with a significantly lower mortality risk regardless tumor location (depth, site), grade, and size (Fig. 3).

### Sensitivity analysis

The characteristics of all the patients with R1 margins (*N*=1,284), including the 255 patients with no RE status available are presented in Supplementary material S1. Assuming the patients with RE status non available as not having been reoperated, the univariate analysis showed that OS was similar in the patients with RE status missing and No-RE patients (Supplementary material S2); in the multivariate analysis, RE remained a favorable prognostic factor for OS after adjustment on major prognosis factors (HR 0.36, 95% 0.23–0.56, *p*<0.0001 (Supplementary material S4).

## Discussion

Our results issued from the French nationwide prospective database NETSARC registering all sarcoma and connective tissue tumors since 2010 show a significantly improved OS, RFS, and LRFS with a median follow-up of 31 months in patients with an initial R1 resection conducted outside of a reference center for a limb or trunk wall soft tissue sarcoma, and RE as part of adjuvant treatment. The benefit of RE on OS is observed in almost all subgroup of patients *i.e.* all other thing being equal, meaning regardless of age, tumor size (deep/superficial seated), location (lower/upper or trunk), grade (1/2 or 3), and histology. Our series is the first and largest so far to our knowledge, using direct comparison of prospectively registered patients with and without RE after a first surgery outside of a reference center, and prompt us to systematically consider RE in patients with potential microscopic margins (R1) initially operated outside a reference center.

Once unplanned resection has been carried out, there is a general consensus in the literature for the need of further resection to remove the potential residual tumor and achieve resection with appropriate margins in order to improve oncologic outcome, i.e. local control and disease specific survival. Most of the authors recommend RE based on the high incidence (31 to 72%) of residual tumor in the re-excision specimen [9, 10, 13, 19–23]. Residual tumor is considered as an unfavorable prognosis factor, with impact on local recurrence [19], but also recurrence free survival, metastasis free survival, and overall survival [5, 9–11, 24], as recently reported in the systematic review of Sacchetti and colleagues [25].

In addition, RE has also been recommended based on equivalent or even better oncologic outcome in patients reexcised after unplanned resection compared with patients with only primary resection. Several subsequent studies showed similar or even better control of local recurrence, metastasis free survival, and survival [9, 13, 26] with re-excision of unplanned first surgery compared with one stage surgery [27–29].

Overall, most studies indirectly support the idea that RE after unplanned resection improves not only local tumor control but also disease-free specific survival whereas some studies questioned the association between RE and OS or distant metastasis, and would consider the option of postponing RE. Meanwhile, Lewis *et al.* reported no correlation between residual tumor on re-excision specimen and disease specific or recurrence free survival in a series of 407 re-excised sarcomas [13]. Recently, Danieli and colleagues also showed that a residual disease in the RE tumor-bed was not associated with higher risk of distant metastasis and lower OS in a large cohort of patients surgically treated from 1997 to 2017 [14], and authors proposed to consider postponing reexcision after macroscopic complete unplanned excision until local recurrence occurs, on a case-by-case basis.

Decanter *et al*. recently investigated systematic RE after unplanned resection of extremities and superficial trunk STS in patients first operated out of reference centers [15], and reported that systematic RE in sarcoma specialized centers offered better local control but did not impact OS. However, results were issued from a different study population including 395 (70%) R0 patients and 168 (29%) R1 patients after first surgery. Indeed, the present study included only confirmed sarcomas with R1 margins after first resection; R2 and unknown margins resections, as well as tumors of intermediate malignancy, atypical lipomatous tumors, dermato-fibrosarcoma protuberans tumors, desmoid tumors and patients with metastasis at diagnosis were excluded. This highly selected population of R1-patients and not all unplanned surgical procedures carried out outside reference centers as usually reported in the whole literature, is particularly appropriate to report RE benefit in patients at higher risk.

The present study does not allow to conclude that all patients with R1 resection initially operated outside reference centers might be re-operated, and better identification of subgroups of patients for whom RE should be recommended, or conversely discouraged, is required. The subgroup analysis conducted in RE patients to address this issue showed similar HR for all subgroups considering tumor depth, location, grade, and size. Nevertheless, exploration in patients with good prognosis (*i.e*. small and superficial and low-grade tumors) was limited by the too reduced sample size of patients and events (disease-related death), and further studies need to focus on this specific topic. So far, RE has to be discussed for all patients after unplanned R1 resection outside of NETSARC center.

In a reference center, R1-margins are mostly anticipated by a pre-treatment decided by MDTB; unexpected R1-margins rarely occur [30]; such cases likely translate tumoral biomarker of aggressiveness [31]. The signification of R1 surgery carried out of a reference center deems different: based on the improvement of LRFS, as well as RFS and OS after RE, R1 status would more likely be considered as a marker of inadequate surgery rather than a marker of aggressiveness or what we retrospectively considered as R1 margins includes in fact some R0 margins’ resections.

There are several limitations in the current study. Firstly, despite prospective data collection, this multicenter retrospective design leads to some selection biases that may affect results: RE decision and to what extent bed tumor should be re-excised is a critical process which is complex to track retrospectively; RE decision does not rely on the same arguments for all patients and all surgeons. The large sample size, the guidelines shared between centers may reduce, but not completely erase this bias. Secondly, we can not exclude that some patients failed to be referred to reference centers by clinicians, or to be registered by pathologist and ultimately missed. Nevertheless, the nationwide incidence of STS suggests that NETSARC network established a closest to exhaustive national collection from 2013 [2]. Thirdly, assessment of R1 margins relying on data from first pathology, surgical, macro- and microscopic analysis and discussion between surgeons and pathologist is a critical issue and increased accuracy would be expected [32]; Notably, uncertainty remains between R1 and R0 in case of thin margins [33], and margin classification outside of reference centers may be questionable. R2 margins-resections are easy to identify and rule out. In case of any doubt between R0 and R1, resection was considered R1. Finally, RE impact on OS, on local and distant recurrence, actually implies to consider the complete adjuvant treatment strategy and surveillance modalities associated with RE process, which were not captured in the present work. Nevertheless little and controversial impact of chemotherapy on oncologic outcome is reported in the literature, and radiotherapy is considered not to impact OS, the primary objective of this study. RE results must be assessed in the light of these consensus statements on adjuvant therapy [3, 8]. Finally, we relied on multivariate analysis to adjust for observable selection bias. A propensity score method confirming significant impact of RE on OS, RFS and LRFS has also been used to control the selection bias despite the literature reviews have reported equivalent results to traditional regression for eliminating the bias on observed variables (supplementary material S3). However, none of these methods consider the bias due to unobserved variables, i.e. not collected in the study [34].

To address the issue of RE after surgery out of a reference center, other nationwide studies from other countries are necessary. In parallel, commitment to continuous quality improvement for extensive data collection must be applied, namely access to reliable data with accurate margin status qualification from any operative and pathology reports will contribute to minimize missing data for patients treated outside of a reference center. Finally, earlier referral of patients prior to any surgery would ensure appropriate quality of information mandatory for more relevant in-depth studies.

In conclusion, the present study highlights the importance of re-excision as part of an “adjuvant” multidisciplinary treatment after R1 margins surgical treatment of a sarcoma of extremities and trunk wall outside of a sarcoma reference center to improve survival and reduce relapse. All subgroups of patients are eligible to discuss RE.

## Supplementary Information

**Additional file 1:** Supplementary material S1, S2, S3 and S4

## Data Availability

The nationwide database NETSARC (netsarc.org) that support the findings of this study contains information that could compromise privacy of the research participants. The anonymised data sets are available upon reasonable request from the data protection officer of the Léon Bérard cancer center at DPD@lyon.unicancer.fr.
