# Supplementary material for: Overall survival in patients with re-excision of positive microscopic margins of limb and trunk wall soft tissue sarcoma operated outside of a reference center: a nationwide cohort analysis
Source: BMC Cancer. 2022 Oct 3;22:1034. doi: 10.1186/s12885-022-10121-5 (PMC9531489; doi:10.1186/s12885-022-10121-5)
Supplement: Supplementary file 1 — Additional file 1: Supplementary material S1, S2, S3 and S4 [file 12885_2022_10121_MOESM1_ESM.docx]

***Supplementary material S1*.** **Characteristics of all patients first operated outside NETSARC reference centers (n=1,284 patients).** Data are mean (SD) or n (%). MDTB: multidisciplinary tumor board. Percentages might not add up to 100% due to rounding. KW: Kruskall-Wallis test

|  | R1 OUTSIDE NETSARC  no RE | | | R1 OUTSIDE NETSARC  RE | | | | | Re-excision  not specified  (RE missing) | | | | | | | Total | | | | | | | Test | | | |  |  |  |  |  |
| --- | --- | --- | --- | --- | --- | --- | --- | --- | --- | --- | --- | --- | --- | --- | --- | --- | --- | --- | --- | --- | --- | --- | --- | --- | --- | --- | --- | --- | --- | --- | --- |
|  | N =331 | | | N =698 | | | | | N =255 | | | | | | | N =1284 | | | | | | |  |  |  |  |  |  |  |  |  |
| Clinical characteristics |  |  | | | |  | |  | | | | |  | |  | | | |  | | |  |  | | | | |  |  |  |  |
| Sex |  |  | | | |  | |  | | | | |  | |  | | | |  | | |  | | Chi-2 P = 0.936 | | | | | |  |  |
| Female | 147 | (44.4%) | 313 | | | | (44.8%) | | | | 117 | | | (45.9%) | | | | 577 | | | (44.9%) | | |  | | | | | |  |  |
| Male | 184 | (55.6%) | 385 | | | | (55.2%) | | | | 138 | | | (54.1%) | | | | 707 | | | (55.1%) | | |  |  |  |  |  |  |  |  |
| Age at diagnosis |  |  |  | | | |  | | | |  | | |  | | | |  | | |  | | | KW P = 0.001 | | | | | |  |  |
| N | 331 | | 698 | | | | | | | 255 | | | | | | | 1284 | | | | | | | |  | | | | | | |
| Missing | 0 | | 0 | | | | | | | 0 | | | | | | | 0 | | | | | | | |  |  |  |  |  |  |  |
| Median (Q1-Q3) | 63 (45-77) | | 61(44-71) | | | | | | | 63(50-77) | | | | | | | 62(45-74) | | | | | | | |  |  |  |  |  |  |  |
| Site of tumour |  |  |  | |  | | | | |  | |  | | | | |  | | |  | | | | | Chi-2 P = 0.007 | | | | | |  |
| Lower limb | 135 | (40.8%) | 310 | | | | (44.4%) | | | | 113 | | | (44.3%) | | | | 558 | | | (43.5%) | | | | |  | | |  |  |  |
| Trunk wall | 134 | (40.5%) | 206 | | | | (29.5%) | | | | 83 | | | (32.5%) | | | | 423 | | | (32.9%) | | | | |  |  |  |  |  |  |
| Upper limb | 62 | (18.7%) | 182 | | | | (26.1%) | | | | 59 | | | (23.1%) | | | | 303 | | | (23.6%) | | | | |  |  |  |  |  |  |
| Size of the tumor (mm). |  |  |  | |  | | | | |  | |  | | | | |  | | |  | | | | | KW P <0.001 | | | | | |  |
| N | 308 | | 669 | | | | | | | 247 | | | | | | | 1224 | | | | | | | |  |  |  |  |  |  |  |
| Missing | 23 | | 29 | | | | | | | 8 | | | | | | | 60 | | | | | | | |  |  |  |  |  |  |  |
| Mean (std) | 72.76 (58.53) | | 47.91 (40.49) | | | | | | | 68.07 (50.86) | | | | | | | 58.24 (49.06) | | | | | | | |  |  |  |  |  |  |  |
| Median (min-max) | 55 (5-500) | | 40 (5-500) | | | | | | | 55 (6-260) | | | | | | | 45 (5-500) | | | | | | | |  |  |  |  |  |  |  |
| Q1-Q3 | 35-92.50 | | 23-60 | | | | | | | 30-90 | | | | | | | 28-73.50 | | | | | | | |  |  |  |  |  |  |  |
| Depth of tumour |  |  |  | |  | | | | |  | |  | | | | |  | | |  | | | | | Chi-2 P <0.001 | | | | | | |
|  | 28 |  | 43 | | | |  | | | | 9 | | |  | | | | 80 | | |  | | | | |  | | |  |  |  |
| Deep | 214 | (70.6%) | 352 | | | | (53.7%) | | | | 179 | | | (72.8%) | | | | 745 | | | (61.9%) | | | | |  |  |  |  |  |  |
| Superficial | 89 | (29.4%) | 303 | | | | (46.3%) | | | | 67 | | | (27.2%) | | | | 459 | | | (38.1%) | | | | |  |  |  |  |  |  |
| Histology |  |  |  | | | |  | | | |  | | |  | | | |  | | |  | | | | | Chi-2 P =0.033 | | |  |  |  |
| Chondrosarcoma | 2 | (0.6%) | 2 | | | | (0.3%) | | | | 0 | | | (0.0%) | | | | 4 | | | (0.3%) | | | | |  |  |  |  |  |  |
| Leiomyosarcoma | 48 | (14.5%) | 160 | | | | (22.9%) | | | | 50 | | | (19.6%) | | | | 258 | | | (20.1%) | | | | |  |  |  |  |  |  |
| Liposarcoma | 62 | (18.7%) | 85 | | | | (12.2%) | | | | 41 | | | (16.1%) | | | | 188 | | | (14.6%) | | | | |  |  |  |  |  |  |
| Malignant peripheral nerve sheath tumour | 14 | (4.2%) | 26 | | | | (3.7%) | | | | 8 | | | (3.1%) | | | | 48 | | | (3.7%) | | | | |  |  |  |  |  |  |
| Miscellaneous sarcomas | 46 | (13.9%) | 76 | | | | (10.9%) | | | | 27 | | | (10.6%) | | | | 149 | | | (11.6%) | | | | |  |  |  |  |  |  |
| Myxofibrosarcoma | 38 | (11.5%) | 115 | | | | (16.5%) | | | | 35 | | | (13.7%) | | | | 188 | | | (14.6%) | | | | |  |  |  |  |  |  |
| Osteosarcoma | 5 | (1.5%) | 13 | | | | (1.9%) | | | | 6 | | | (2.4%) | | | | 24 | | | (1.9%) | | | | |  |  |  |  |  |  |
| Other sarcomas | 5 | (1.5%) | 19 | | | | (2.7%) | | | | 12 | | | (4.7%) | | | | 36 | | | (2.8%) | | | | |  |  |  |  |  |  |
| Rhabdomyosarcoma | 7 | (2.1%) | 12 | | | | (1.7%) | | | | 6 | | | (2.4%) | | | | 25 | | | (1.9%) | | | | |  |  |  |  |  |  |
| Sarcoma | 4 | (1.2%) | 3 | | | | (0.4%) | | | | 5 | | | (2.0%) | | | | 12 | | | (0.9%) | | | | |  |  |  |  |  |  |
| Suspicion of sarcoma | 1 | (0.3%) | 2 | | | | (0.3%) | | | | 2 | | | (0.8%) | | | | 5 | | | (0.4%) | | | | |  |  |  |  |  |  |
| Synovial sarcoma | 21 | (6.3%) | 32 | | | | (4.6%) | | | | 11 | | | (4.3%) | | | | 64 | | | (5.0%) | | | | |  |  |  |  |  |  |
| Undifferentiated pleomorphic sarcoma | 78 | (23.6%) | 153 | | | | (21.9%) | | | | 52 | | | (20.4%) | | | | 283 | | | (22.0%) | | | | |  |  |  |  |  |  |
| Histology |  |  |  | | | |  | | | |  | | |  | | | |  | | |  | | | | | Chi-2 P =0.010 | | |  |  |  |
| Leiomyosarcoma | 48 | (14.5%) | 160 | | | | (22.9%) | | | | 50 | | | (19.6%) | | | | 258 | | | (20.1%) | | | | |  |  |  |  |  |  |
| Liposarcoma | 62 | (18.7%) | 85 | | | | (12.2%) | | | | 41 | | | (16.1%) | | | | 188 | | | (14.6%) | | | | |  |  |  |  |  |  |
| Miscellaneous sarcomas | 46 | (13.9%) | 76 | | | | (10.9%) | | | | 27 | | | (10.6%) | | | | 149 | | | (11.6%) | | | | |  |  |  |  |  |  |
| Myxofibrosarcoma | 38 | (11.5%) | 115 | | | | (16.5%) | | | | 35 | | | (13.7%) | | | | 188 | | | (14.6%) | | | | |  |  |  |  |  |  |
| Other | 38 | (11.5%) | 77 | | | | (11.0%) | | | | 39 | | | (15.3%) | | | | 154 | | | (12.0%) | | | | |  |  |  |  |  |  |
| Synovial sarcoma | 21 | (6.3%) | 32 | | | | (4.6%) | | | | 11 | | | (4.3%) | | | | 64 | | | (5.0%) | | | | |  |  |  |  |  |  |
| Undifferentiated sarcoma | 78 | (23.6%) | 153 | | | | (21.9%) | | | | 52 | | | (20.4%) | | | | 283 | | | (22.0%) | | | | |  |  |  |  |  |  |
| Grade of tumour |  |  |  | | | |  | | | |  | | |  | | | |  | | |  | | | | | Chi-2 P =0.203 | | |  |  |  |
| . | 35 |  | 42 | | | |  | | | | 27 | | |  | | | | 104 | | |  | | | | |  |  |  |  |  |  |
| 1 | 43 | (14.5%) | 101 | | | | (15.4%) | | | | 36 | | | (15.8%) | | | | 180 | | | (15.3%) | | | | |  |  |  |  |  |  |
| 2 | 81 | (27.4%) | 227 | | | | (34.6%) | | | | 62 | | | (27.2%) | | | | 370 | | | (31.4%) | | | | |  |  |  |  |  |  |
| 3 | 98 | (33.1%) | 193 | | | | (29.4%) | | | | 76 | | | (33.3%) | | | | 367 | | | (31.1%) | | | | |  |  |  |  |  |  |
| Non dimmable | 74 | (25.0%) | 135 | | | | (20.6%) | | | | 54 | | | (23.7%) | | | | 263 | | | (22.3%) | | | | |  |  |  |  |  |  |
| Patient management |  |  |  | | | |  | | | |  | | |  | | | |  | | |  | | | | |  | | |  |  |  |
| MDTB before treatment |  |  |  | | | |  | | | |  | | |  | | | |  | | |  | | | | | Chi-2 P =0.004 | | |  |  |  |
| No | 286 | (86.4%) | 642 | | | | (92.0%) | | | | 219 | | | (85.9%) | | | | 1147 | | | (89.3%) | | | | |  |  |  |  |  |  |
| Yes | 45 | (13.6%) | 56 | | | | (8.0%) | | | | 36 | | | (14.1%) | | | | 137 | | | (10.7%) | | | | |  |  |  |  |  |  |
| Re-exicision |  |  |  | | | |  | | | |  | | |  | | | |  | | |  | | | | |  | | |  |  |  |
| NA | 331 | (100.0%) | 0 | | | | (0.0%) | | | | 0 | | | (0.0%) | | | | 331 | | | (25.8%) | | | | |  |  |  |  |  |  |
| Inside NETSARC | 0 | (0.0%) | 490 | | | | (70.2%) | | | | 0 | | | (0.0%) | | | | 490 | | | (38.2%) | | | | |  |  |  |  |  |  |
| Outside NETSARC | 0 | (0.0%) | 175 | | | | (25.1%) | | | | 0 | | | (0.0%) | | | | 175 | | | (13.6%) | | | | |  |  |  |  |  |  |
| Unknown | 0 | (0.0%) | 33 | | | | (4.7%) | | | | 255 | | | (100.0%) | | | | 288 | | | (22.4%) | | | | |  |  |  |  |  |  |
| Quality of final surgery |  |  |  | | | |  | | | |  | | |  | | | |  | | |  | | | | | Chi-2 P <0.001 | | |  |  |  |
|  | 0 |  | 38 | | | |  | | | | 255 | | |  | | | | 293 | | |  | | | | |  |  |  |  |  |  |
| Margin not evaluable | 0 | (0.0%) | 2 | | | | (0.3%) | | | | 0 | | | - | | | | 2 | | | (0.2%) | | | | |  |  |  |  |  |  |
| R0 | 0 | (0.0%) | 599 | | | | (90.8%) | | | | 0 | | | - | | | | 599 | | | (60.4%) | | | | |  |  |  |  |  |  |
| R1 | 331 | (100.0%) | 42 | | | | (6.4%) | | | | 0 | | | - | | | | 373 | | | (37.6%) | | | | |  |  |  |  |  |  |
| Unknown | 0 | (0.0%) | 17 | | | | (2.6%) | | | | 0 | | | - | | | | 17 | | | (1.7%) | | | | |  |  |  |  |  |  |

***Supplementary material S2.* Overall survival in patients with R1 resection outside NETSARC centers, including patients with second resection (RE), patients with no second resection (NoRE), and patients with no resection data (missing data, n=255) (n=1,284 patients).**


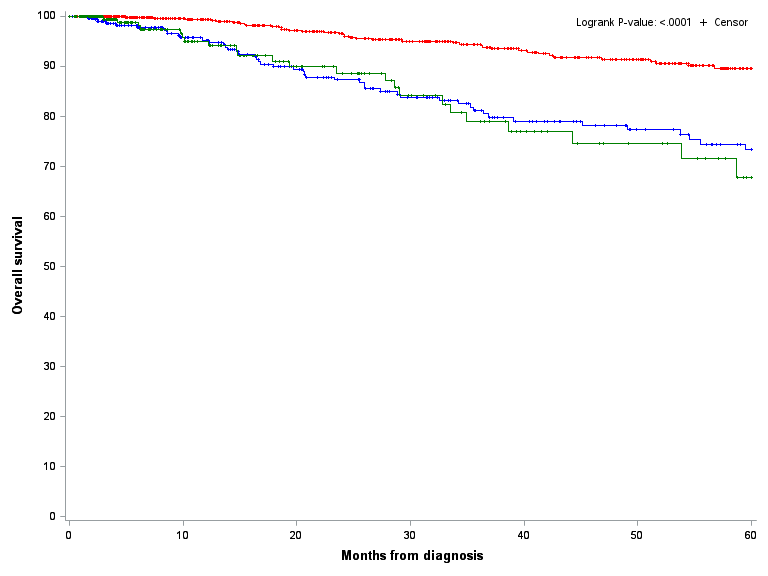


R1 patients -RE

R1 patients -No-RE

R1 patients -RE data missing

***Supplementary material S3.***

**Propensity score matching analysis**: after multiple imputations for handling missing covariates, we implemented the inverse probability of treatment weighting (IPTW), a method using the propensity score to control the potential selection bias associated with nonrandomization. The propensity score was the conditional probability for a patient to be re-excised, conditionally to observable characteristics: age, gender, site of tumor, size of tumor, depth of tumor, histology, grade, MDTB before treatment.

Results displayed on the following graphs confirmed the previously observed better oncologic outcome in patients with re-excision. A) OS; B) DFR; C) Cumulative incidence functions (CIR); (p<0.0001 for the 3 outcome measures)

A)


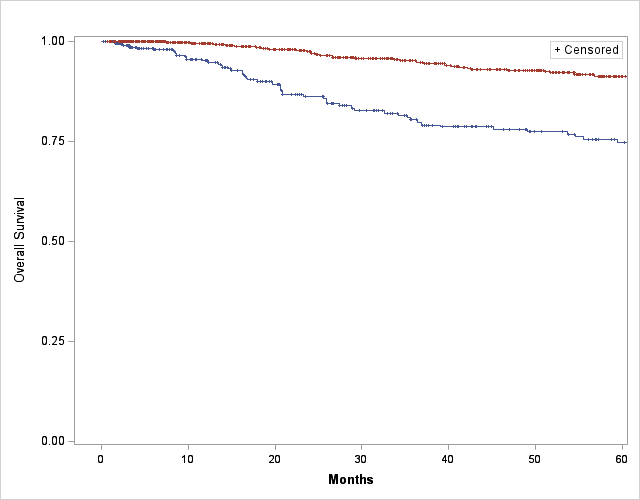

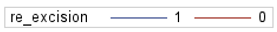


B)


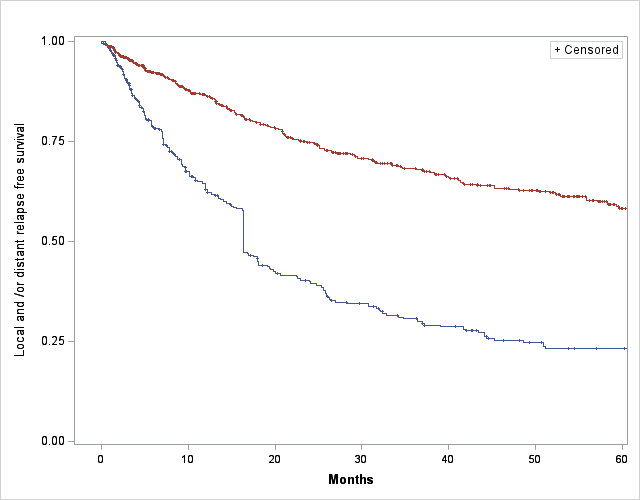

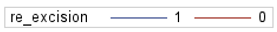


C)


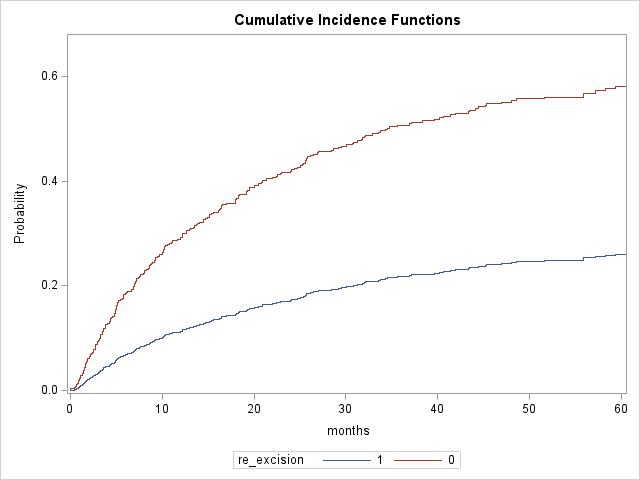


***Supplementary material S4*. Sensitivity analysis in patients with R1 margins operated outside NETSARC centers assuming patients with no RE status available (n=255) as not having been reoperated (n=1,284 patients). Univariate and multivariate analysis for overall survival.** HR: Hazard ratio (95%CI); *p* value; MDBT: multidisciplinary tumor board.

|  | **Overall survival** | |
| --- | --- | --- |
|  | **Unadjusted HR** | **Adjusted HR** |
| Age at diagnosis | 1.03 (1.02–1.04); **<.0001** | 1.03 (1.01–1.04); 0.00 |
| Gender female (ref: male) | 0.83 (0.55–1.26); 0.39 | 0.79 (0.50–1.23); 0.29 |
| Size of the tumor (mm) | 1.01 (1.00–1.01); **<.0001** | 1.00 (1.00–1.01); 0.01 |
| Site of tumor |  |  |
| Trunk wall (ref: lower limb) | 1.14 (0.74–1.77); 0.55 | 0.99 (0.62–1.58); 0.95 |
| Upper limb (ref: lower limb) | 0.44 (0.23–0.86); 0.02 | 0.48 (0.24–0.96); 0.04 |
| Depth of tumor (ref: superficial) | 1.14 (0.74–1.76); 0.56 | 0.84 (0.51–1.37); 0.48 |
| Grade |  |  |
| Grade 3 (ref: grades 1–2) | 2.27 (1.43–3.62); 0.00 | 1.80 (1.09–2.96); 0.02 |
| Non dimmable (ref: grades 1–2) | 1.01 (0.56–1.83); 0.96 | 0.69 (0.24–1.99); 0.49 |
| MDTB before treatment (ref: no) | 1.27 (0.70–2.32); 0.44 | 1.43 (0.74–2.74); 0.29 |
| Histology |  |  |
| Leiomyosarcoma (ref: other) | 0.38 (0.16–0.91); 0.03 | 0.26 (0.09–0.80); 0.02 |
| Liposarcoma (ref: other) | 0.58 (0.26–1.29); 0.17 | 0.22 (0.08–0.63); 0.00 |
| Miscelaneous sarcomas (ref: other) | 0.65 (0.29–1.45); 0.29 | 0.69 (0.25–1.88); 0.47 |
| Myxofibro sarcoma (ref: other) | 0.70 (0.32–1.54); 0.38 | 0.35 (0.12–0.98); 0.04 |
| Synovial sarcoma (ref: other) | 0.78 (0.30–2.05); 0.61 | 0.95 (0.29–3.15); 0.93 |
| Undiffferentiated sarcoma (ref: other) | 1.35 (0.71–2.60); 0.36 | 0.66 (0.27–1.63); 0.37 |
| Re–excision (ref: no) | 0.33 (0.22–0.50); **<.0001** | 0.36 (0.23–0.56); **<.0001** |
